# Editorial Brevities

**Published:** 1887-01-01

**Authors:** 


### EDITORIALS AND MISCELLANEOUS.

### EDITORIAL BREVITIES.

THE RECORD will hereafter be punctually mailed to its patrons on the first day of each month, unless Providentially prevented. The present number, and all numbers of the present volume, will contain four additional pages of reading matter. Our pages are large and compact, and the matter of our journal will be found condensed, varied and practical. ☞ Those of our friends who are in arrears will please remit promptly, and all are requested to renew their subscriptions. We trust that all will do so at once, and that more of them will avail themselves of our liberal offer to accept $3 for two copies on renewal. Every one can, by a little effort, induce a medical friend to take the RECORD and thus secure reduced rates for the next year.

THE BANDAGE AFTER DELIVERY.—It appears that considerable discussion ls going on as to the use of the obstetric binder after delivery. The weight of the argument, we think, is in favor of the binder, to be worn some weeks after child-birth, to maintain the integrity of the abdominal muscles and to prevent the pendulous belly so often observed in the child-bearing woman.

THE IVY STREET HOSPITAL is nearing completion. When finished, it will be a great boon to the poor of Atlanta—a most suitable adjunct to the Southern Medical College—a monument to the benevolence of the noble Women of the Gate City and to the sagacity and indomitable energy of Dr. T. S. Powell, the Superintendent. W.

THE SOUTHERN MEDICAL COLLEGE is in a most flourishing condition. Each session opens up with increasing popularity. The Class of 1886–'7 is the largest that has ever attended this Institution. New Chairs have been founded ; new appliances have been introduced ; greater clinical facilities have been developed ; the Hospital has been greatly enlarged. The College is destined to soon become the strongest center of Medical Education in the South. It has students from all parts of the Union. North, South, East and West are well represented.

DEATH OF DR. KINNEBREW—Dr. Henry Kinnebrew, of Clarke County, Ga., recently dropped dead in his buggy while talking to a friend. The sad event occurred about three miles below Athens. Dr. Kinnebrew practiced his profession in Clarke and Oglethorpe counties, and was in the sixty-third year of his age.

SURGICAL AND GYNECOLOGICAL ASSOCIATION.—An association of the above character was, we learn, organized in Alabama on the 15th ultimo. We have not yet been furnished with the officers elect, etc., of the new association, the starting of which, in Alabama, is indicative of medical progress in that State.

WE are informed that Drs. Taliaferro & Noble have just removed their Infirmary to St. Joseph Hospital.

ATLANTA is emphatically the Paradise of City Ward Physicians. Some of them manage to keep body, soul and horse together on a salary amounting to less than a dollar a day!

GYNECOLOGICAL SPARRING.—There has been, of late, some unpleasant sparring between the two well-known gynecologists, Dr. Robert Battey and Mr. Lawson Tait. So far, the discussion has been carried on through the medical press, on both sides of the Atlantic. We hope that, at the International Congress, we will find the two gentlemen *tete a tete.*

DR. K. C. DIVINE went to Florida on a prospective tour. No sooner did he disembark in the Land of Flowers and Oranges than he returned, satisfied that Atlanta was the place after all. He should have *divined* that before he went.

MY EYES! A journalistic cotemporary has indiscreetly published that there is an oculist in Atlanta who collects $2,000 monthly! Great Scott! The amount of eye doctors that this startling revelation is going to bring to the Gate City we anticipate to be simply fabulous.

OFFICERS ELECT OF ATLANTA MEDICAL SOCIETY.—At the annual meeting of the Atlanta Society of Medicine, held on Tuesday evening, December 21, the following officers were elected for the year 1887: President, Dr. Virgil O. Hardon; Vice-president, Dr. Henry Wile; Recording Secretary, Dr. H. W. McRae; Corresponding Secretary, Dr. W. S. Elkin; Treasurer, Dr. Hunter P. Cooper; Reporters, Dr. James A. Gray, Dr. W. F. Westmoreland, jr. and Dr. H. F. Scott.

MEDICAL ASSOCIATION OF GEORGIA.—It is well to remind those who are appointed to prepare papers for the Medical Association of Georgia that the meeting takes place on the third Wednesday in April next, in Atlanta.

SUPERIOR CARMINATIVE.—Under the head of "Practical Notes and Formulæ," in the present issue, will be found a prescription with the above caption, which is worth to every subscriber more than the yearly subscription price of the RECORD; and, as some return for so valuable a suggestion, we ask every reader who has a special hobby of his own, which, in practice, he has found to be especially useful, to send it to us, that we may give it a place in our journal. Please don't forget this request.

SURGEON-GENERAL OF THE U. S.—Lieutenant-Colonel John Moore, who has been Acting Medical Purveyor at San Francisco, has received from President Cleveland the appointment of Surgeon-General of the United States. Surgeon-General Moore is a native of Indiana, and has been in the service since 1853.

MESSRS. PARKE, DAVIS & CO.—We are pleased to acknowledge a beautiful card, with the compliments of the Season, from that excellent and reliable business house of Parke, Davis & Co., Detroit, Mich. Their advertisement, on the outside cover of this journal, will always be found to contain something interesting and useful.

WM. R. WARNER & CO.—We thankfully acknowledge the receipt of a beautiful card extending the greetings of the season from another one of our esteemed patrons, Wm. R. Warner & Co., of Philadelphia. This excellent and reliable business house keeps constantly in the RECORD a very interesting advertisement which will well repay perusal.

DR. BANKS PLEDGER has removed from Atlanta to Acworth, Ga. Dr. P. is a graduate of the Southern Medical College, and took the first honor in Obstetrics and Diseases of Women and Children at his final examination. He is a gentleman of good moral character and worthy of the coufidence of the people in his new field of labor.

DR. EDWARDS, until recently a practitioner of this city, has removed to Birmingham, Ala. He is a young man of fine attainments, and of several years' successful experience in his profession. We take pleasure in saying that he is a good physician, and we doubt not will sustain himself with credit in the city of Birmingham.

NEW ADVERTISEMENTS.—Attention is invited to the following new advertisements in this issue :

Oleochyle, from the house of George W. Laird & Co.
Bellvue Hospital Chemical College.
Provident Medical Works, St. Louis.
Vin. Mariana Coca, New York.
George R. Hopkins & Co., St. Louis.
R. L. Palmer, Atlanta, Ga.

THE COLUMBIA BICYCLE CALENDAR FOR 1887.—The Columbia Bicycle Calender for 1887, just issued by the Pope Manufacturing Company, of Boston, is a truly artistic and elegant work in chromo-lithography and the letter-press. Each day of the year appears upon a separate slip, with a quotation pertaining to 'cycling from leading publications and prominent personages. The notable 'cycling events are given ; and concise opinions of the highest medical authorities ; words from practical wheelmen, including clergymen and other professional gentlemen ; the rights of 'cyclers upon the roads ; general wheeling statistics ; the benefit of tricycling for ladies ; extracts from 'cycling poems, and much other information interesting alike to the 'cycler and to the general reader. In fact, it is in minature a virtual encyclopædia upon this universally utilized modern steed.

